# Supplementary material for: Genome scale metabolic network modelling for metabolic profile predictions
Source: PLoS Comput Biol. 2024 Feb 22;20(2):e1011381. doi: 10.1371/journal.pcbi.1011381 (PMC10914266; doi:10.1371/journal.pcbi.1011381)
Supplement: S1 Text — Fig A: Reproduction of Fig 3 from Thiele et al. 2013 using FVA (original matlab code from the study) and the random sampling method used in SAMBA. Fig B: Venn diagrams of FVA (orange) and sampling (purple) predictions using Recon 2. Fig C: Running means, trace plots and PSRF plots for 3 random exchange reaction fluxes using 100, 10 000 and 100 000 samples, with 3 independent runs for each. Fig D: Flux bounds and distributions for urate and hypoxanthine exchange reactions in Human1 when affected by Xanthinuria Type I, for different flux range reduction values. Fig E: Z-score vs other metric-based rankings for SCD. Fig F: Heatmap based on Fig A in S1 Text, using predicted ranks instead of z-scores. Fig G: Distribution of metabolite z-scores for SCD. Fig H: Distributions of FVA bound differences. Fig I. Distances from the top 50 most changed metabolites to each of the reactions affected by the SCD SNP. Fig J: Undirected subnetwork showing paths between the reactions affected by SCD and the top 50 predicted most changed metabolites for this condition. Fig K: ChemRich using top 54 metabolite sampling predictions for Fish-eye disease/ LCAT deficiency. Table A: Table of the top 10 most differentially changed metabolites for the SCD gene KO using SAMBA. Table B: ChemRich provides a metabolite-level table with each metabolite assigned to a cluster, the top 10 of which are shown in S2 Table. (PDF) [file pcbi.1011381.s001.pdf]

Supporting information S1 Text

Fig A in S1 Text. Reproduction of Figure 3 from Thiele *et al.* 2013 using (A) FVA (original matlab code from the study) and (B) the random sampling method used in SAMBA.

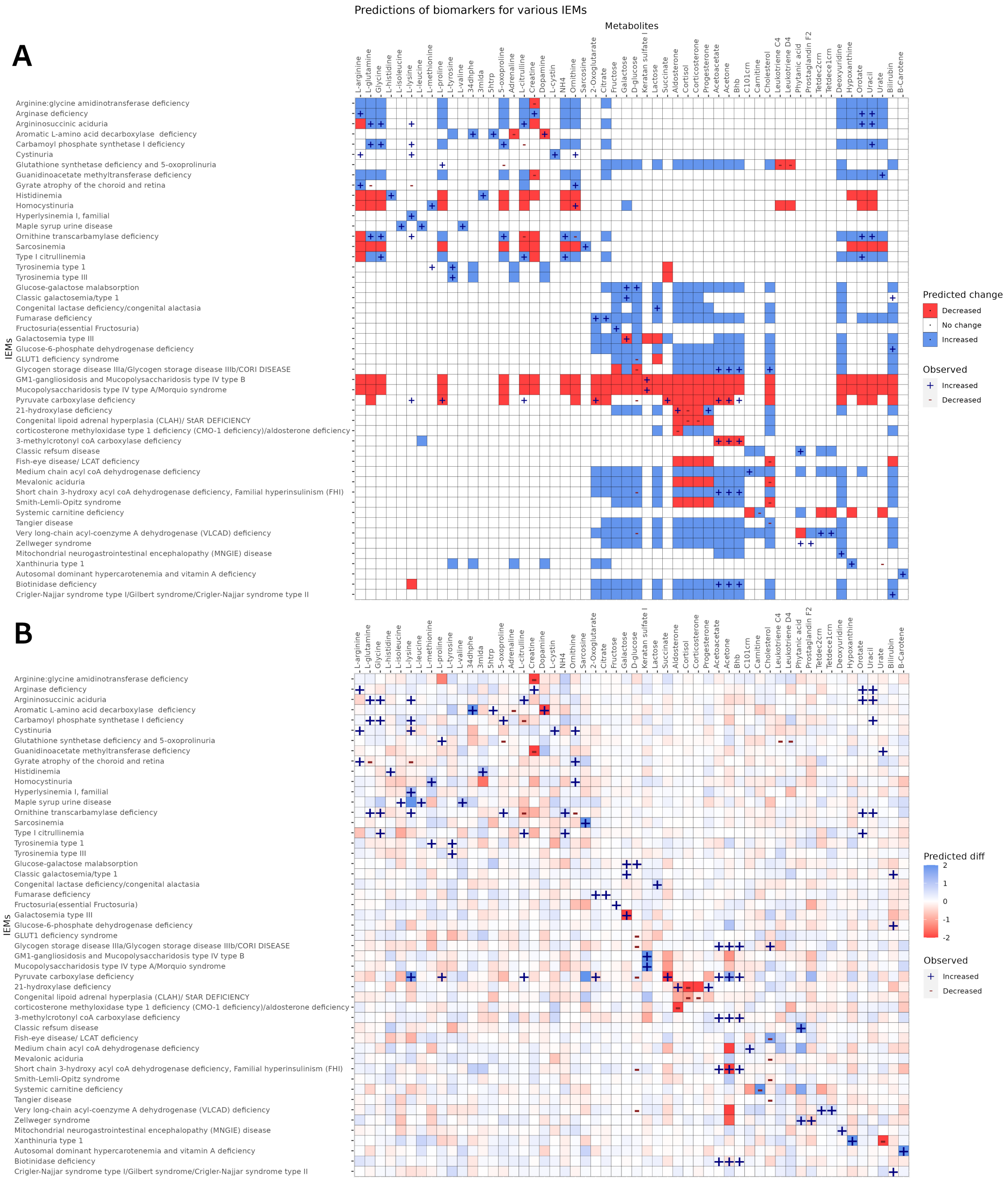

The sampling results, due to the use of no cut-off, show a gradient of z-scores which is more difficult to evaluate systemically than the previous binary results. Despite this, some observed biomarkers are correctly predicted, while others are not. This view of the metabolite predictions also highlights the scale of changes as opposed to the binary "on/off" changes shown in previous heatmaps.

Fig B in S1 Text. Venn diagrams of FVA (orange) and sampling (purple) predictions using Recon 2.

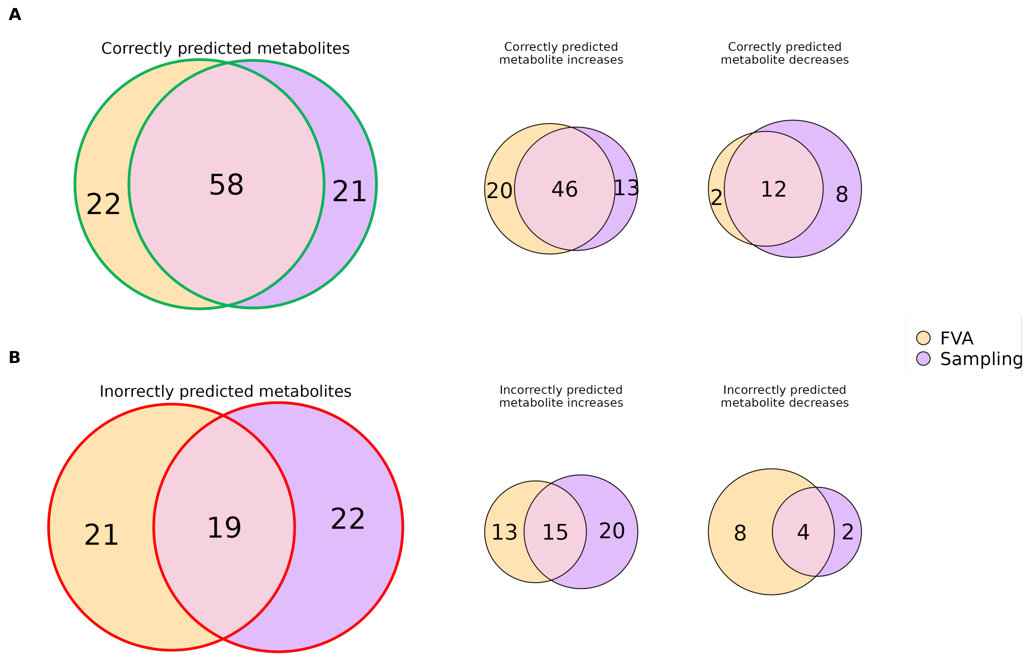

Fig B in S1 Text shows the overlap between various predictions of the IEM subset using FVA and sampling. In orange, the FVA predictions are classed as correct or incorrect depending on the expected increase, decrease or no change. In purple, sampling was run on the same model with the same reaction KOs and model parameters. No z-score threshold was used, meaning that no metabolite is truly classed as "no change" for sampling, and only the prediction of directionality was taken into account. A correctly predicted increase (decrease) is a predicted increase (decrease) where the IEM subset reports an observed increase (decrease respectively). An incorrect increase or decrease is the sum of (i) predictions of the incorrect change direction (for FVA and sampling) and (ii) missing predictions where an IEM change is observed (for FVA).

Regardless of prediction change direction, FVA and sampling both predict a similar number of correct and incorrect metabolite changes, with each method predicting new metabolite changes not predicted by the other. This comparison highlights the difficulties in comparing the differences in each method, as FVA is used with a binary conversion to increase/decrease/no change, whereas with sampling a gradient of scores highlights the changes in many metabolites at once. More

specifically, while the predictions of the two methods overlap frequently, sampling predicts metabolite decreases better than FVA: 20 correct predictions vs 14 for FVA, and 6 incorrect predictions vs 12 for FVA, but predicts fewer metabolite increases correctly.

Fig C in S1 Text. Running means, trace plots and PSRF plots for 3 random exchange reaction fluxes using 100 (A), 10 000 (B) and 100 000 (C) samples, with 3 independent runs for each.

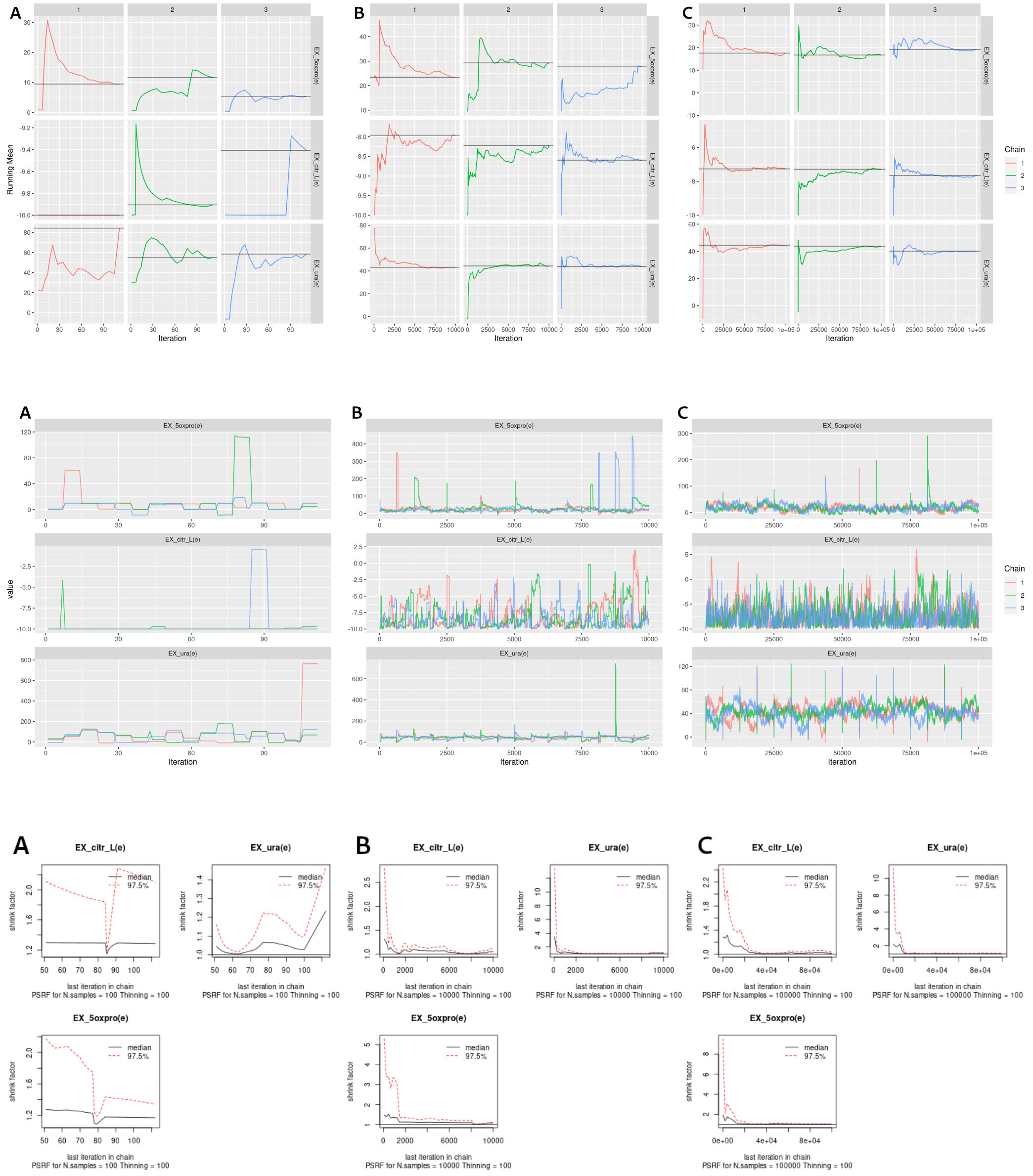

We ran convergence tests using various well-known sampling metrics: running means, traceplots, and shrink factor or PSRF (potential scale reduction factor) plots to make sure that using 100 000 samples was enough for a network this large, with the goal of calculating z-scores on distributions. The results can be found in the following figures, where three independent runs for each nsamples value (100, 10 000, and 100 000) are shown for three randomly chosen exchange reactions in the network.

Running means show how the cumulative average value of all sampled fluxes converges with each sampling iteration. If the final value (black horizontal line) is different between the 3 independent runs (columns per subplot), this shows that there were not enough iterations to reach convergence. The running means clearly show the variability between runs (red, green and blue) when using 100 samples, and still show some variability with 10 000 samples. The running means for 100 000 samples show convergence in each run as well as inter-run stability.

Trace plots show the flux value (y-axis) along iterations (x-axis), for each independent run. Generally, traceplots should show no general trend if there is convergence. For all three nsamples values the trace plots appear relatively stable. For nsamples = 100, the spikes appear larger due to the smaller number of iterations, but these spikes in flux values are normal, even for high nsamples values, as they represent an extreme flux value being found as a solution.

PSRF plots show the shrink factor along iterations. It indicates if runs have "forgotten" their initial flux value. It should decline to 1 as the iterations approach infinity. The PSRF plots for 100 samples have clearly not converged to 1, whereas those for 10 000 and 100 000 samples rapidly approach 1.

Fig D in S1 Text. Flux bounds and distributions for urate and hypoxanthine exchange reactions in Human1 when affected by Xanthinuria Type I, for different flux range reduction values. The top plot row (0) corresponds to the full KO state. A knock-down of 0.1 corresponds to the MUT state reactions only having 10% of their maximum flux range. Flux values are shown on a log2(value+2) scale.

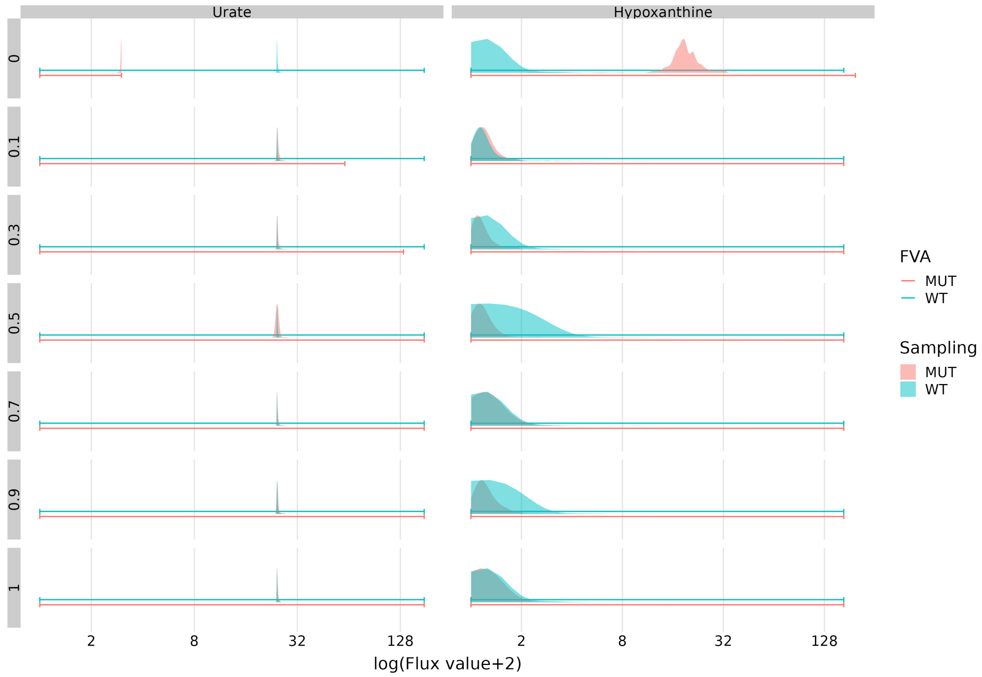

In addition to completely knocking out reaction fluxes, they can be instead partially reduced using a new method developed for this thesis project. The reaction KD method is the following: instead of completely blocking one or several reaction(s), the MUT state can consist of a reduction percentage of the maximum possible fluxes for a given set of reactions. This is done by first calculating the maximum possible flux range using FVA in the chosen metabolic condition. Then, depending on if the FVA upper bound ub and lower bound lb are forward, backward or reversible, the flux reduction is carried out differently:

$$
\begin{aligned}
&ub_{new} = lb_{FVA} + (r * (ub_{FVA} - lb_{FVA})) \text{ for forward reactions } ([0, 1000]) \\
&lb_{new} = ub_{FVA} + (r * (lb_{FVA} - ub_{FVA})) \text{ for backward reactions } ([-1000, 0]) \\
&\begin{cases} ub_{new} = ub_{FVA} * r \\ lb_{new} = lb_{FVA} * r \end{cases} \text{ for reversible reactions } ([-1000, 1000])
\end{aligned}
\tag{1}
$$

By using this reduction strategy, each total interval space is reduced to $r*$ total range. For example, an interval of $[-1000, 1000]$ has a total range of 2000, therefore to reduce the range to 30% of 2000 (600), both bounds must be multiplied by 0.3, leaving us with $[-300, 300]$ and a total range of 600.

The result of reducing fluxes instead of fully knocking them out is that exchange reactions are affected to a lesser extent. The two examples shown in Fig D in S1 Text show two different levels of disruption due to knock downs, using the example from the main text for Xanthinuria Type I.

In the MUT 0% flux state, both urate and hypoxanthine are affected in the most ”extreme” manner possible, shown by the FVA bound differences and the sampling distributions shifts in the top plots of Fig D in S1 Text. However, as the flux reduction percentage increases, meaning less

extreme knock downs, the changes are less drastic: in both cases, the distributions do not shift for any reduction value. For urate, the FVA bounds are reduced to a lesser extent (compared with the 0% state) for 10% and 30% flux reduction. Hypoxanthine is not affected regardless of the reduction value.

Fig E in S1 Text. Z-score vs other metric-based rankings for SCD. In each subplot, the z-score rank is on the left with the same total order of metabolites. On the right of each subplot is a different ranking generated using a different scoring metric, such as the ratio between the means of the two distributions for each metabolite. The metabolites highlighted in red are those experimentally observed in the mGWAS study for SCD.

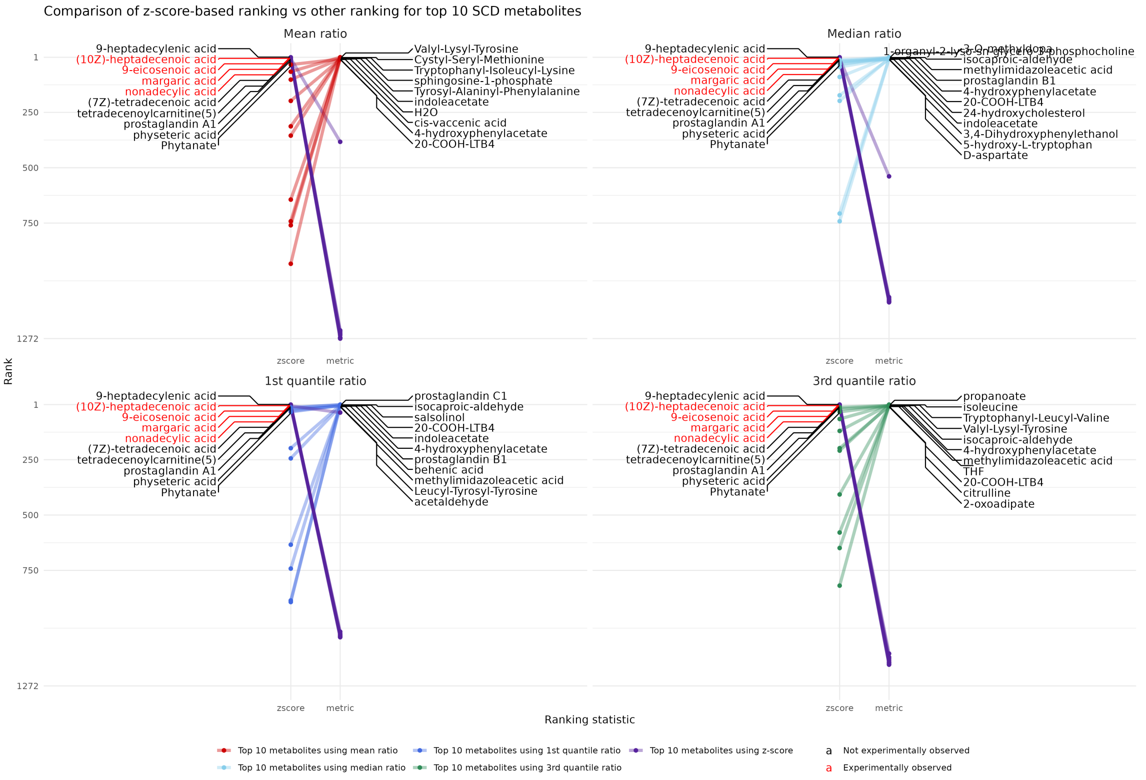

When comparing differences (Fig 4 in main text) and ratios (Fig E in S1 Text), subtracting means or medians appears to be better than using a ratio, which is akin to a fold change. Indeed, using fold changes in this scenario can be limiting due to its bias towards values close to zero. Both the mean and median differences result in a similar top 10 and rank the z-score top 10 metabolites below the 200 mark, whereas when using ratios, no experimentally observed metabolites are highly rankd and the z-score top 10 are poorly ranked.

Fig F in S1 Text. Heatmap based on Fig A in S1 Text, using predicted ranks instead of z-scores.

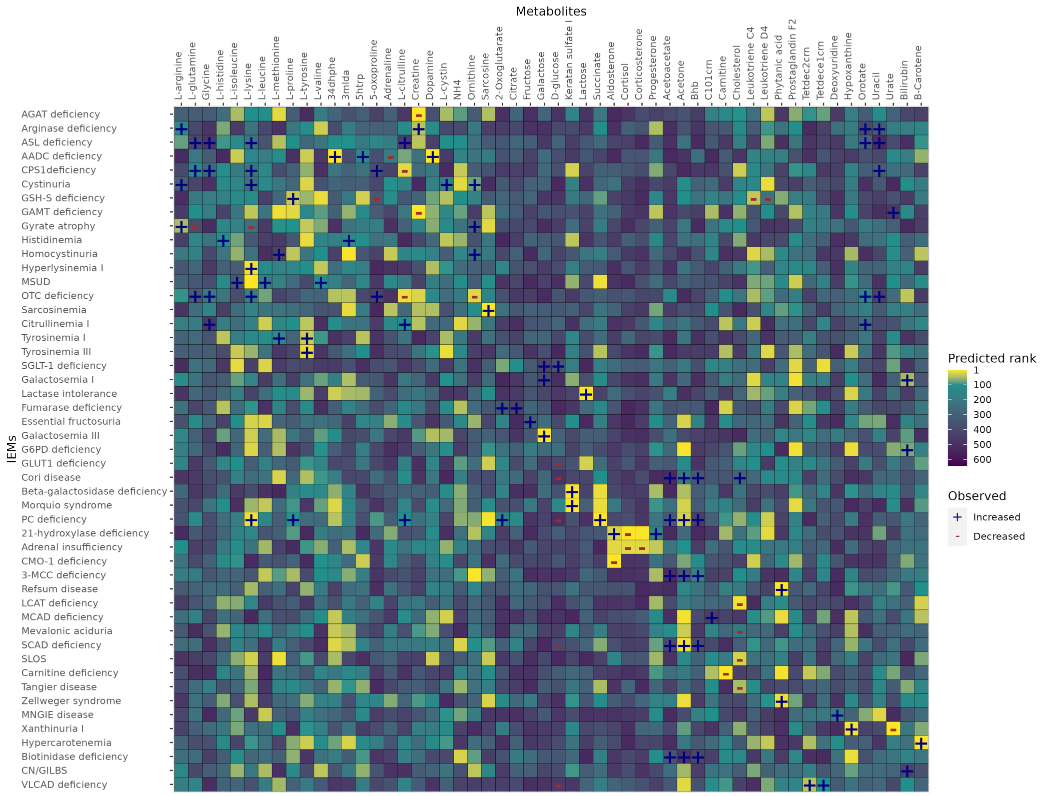

A different method of displaying the sampling results is to plot the ranks directly on the heatmap instead of the z-scores as in Fig A in S1 Text. The ranks are calculated by ordering metabolites by decreasing absolute value of z-scores, which results in the most deregulated metabolites, regardless of direction, being at the top of the ranked list. This of course causes the loss of information on the direction of each change but can serve to quickly highlight the most deregulated metabolites.

Fig G in S1 Text. Distribution of metabolite z-scores for SCD. The metabolite labels highlighted in red are significantly observed in the Suhre *et al.* paper. Here, a threshold of 1 is used to show the metabolites that pass the threshold (blue).

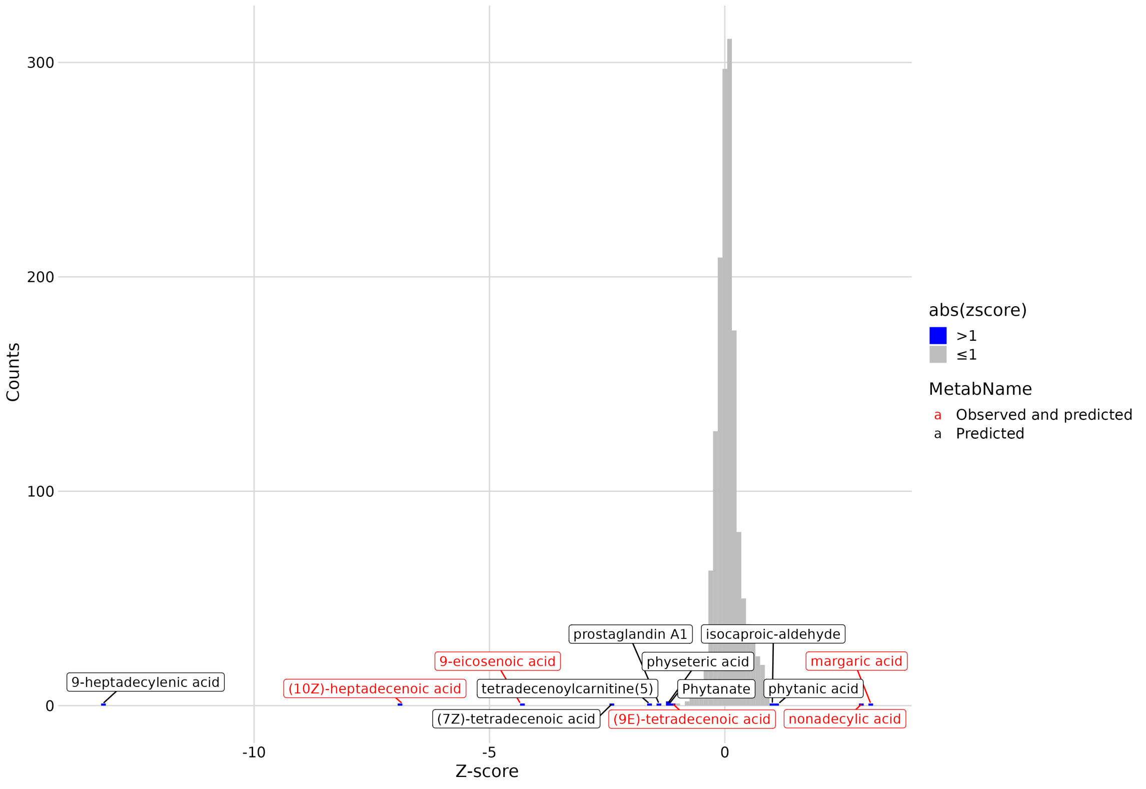

Metabolite ranks are determined from the highest absolute values of z-scores for each metabolite among the entire list of metabolite changes for that condition. This means that the top best ranked metabolites are relative to the rest of the list. Generally, the top most changed metabolites have "extreme" z-score values due to large shifts in distributions caused by the metabolic perturbation. In the SCD example, Fig G in S1 Text shows the distribution of z-scores for all metabolite predictions. Those with a z-score higher than 1 or lower than -1 are highlighted in blue (threshold chosen for illustrative purposes), and the expected metabolite names predicted in the top 10 are shown in red.

This distribution shows how the few extreme z-scores are different to the main "body" of the distribution, and highlights the fact that differences between low ranks are very small in terms of z-score values. Most z-scores are very close to 0 meaning that the rank at this level does not hold much value when comparing between two low ranks. This of course must be taken into consideration when interpreting these simulated metabolic profiles.

Fig H in S1 Text. Distributions of FVA bound differences. Lower bound differences (top panel) and upper bound differences (bottom panel) between WT and MUT for 5 IEMs, for all metabolite exchange reactions. The metabolite labels highlighted in red are significantly observed in patients with the corresponding IEM.

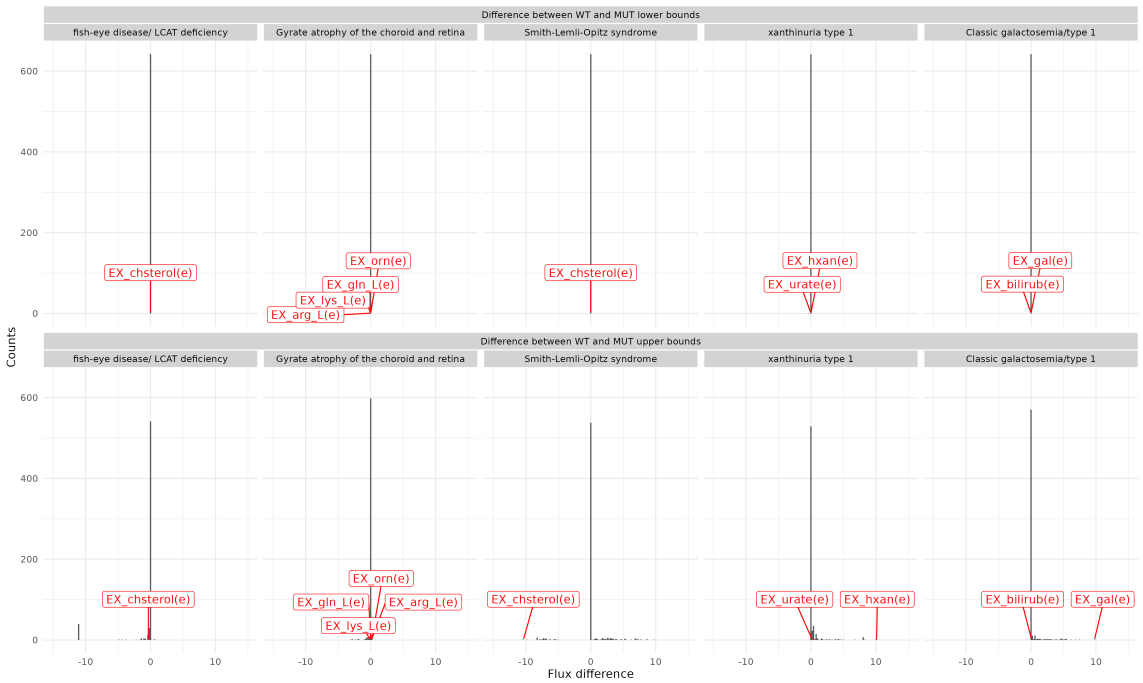

These figures show that the differences between MUT and WT FVA bounds are often close to 0, and are not normally distributed. Sometimes the expected metabolite is an extreme change (and therefore predicted correctly) while in other cases it is close to zero and among a list of other changed metabolites. This demonstrates that a ranking metric or threshold would not be feasible on this type of data due to the almost binary nature of the results: either at or close to zero, or an extreme shift in bounds. Z-scores (see Fig G in S1 Text) follow a normal distribution meaning a threshold and ranking metric is more coherent since the scores are on a continuous scale.

Fig I in S1 Text. Distances from the top 50 most changed metabolites to each of the reactions affected by the SCD SNP. Ranks are shown from beige to dark purple. Distance is shown on the yellow to dark blue-green scale (black is infinite distance, i.e. no path). The distance is measured in the number of reactions and metabolites it takes to get from the KO'd reaction to the extracellular metabolites, using the shortest paths.

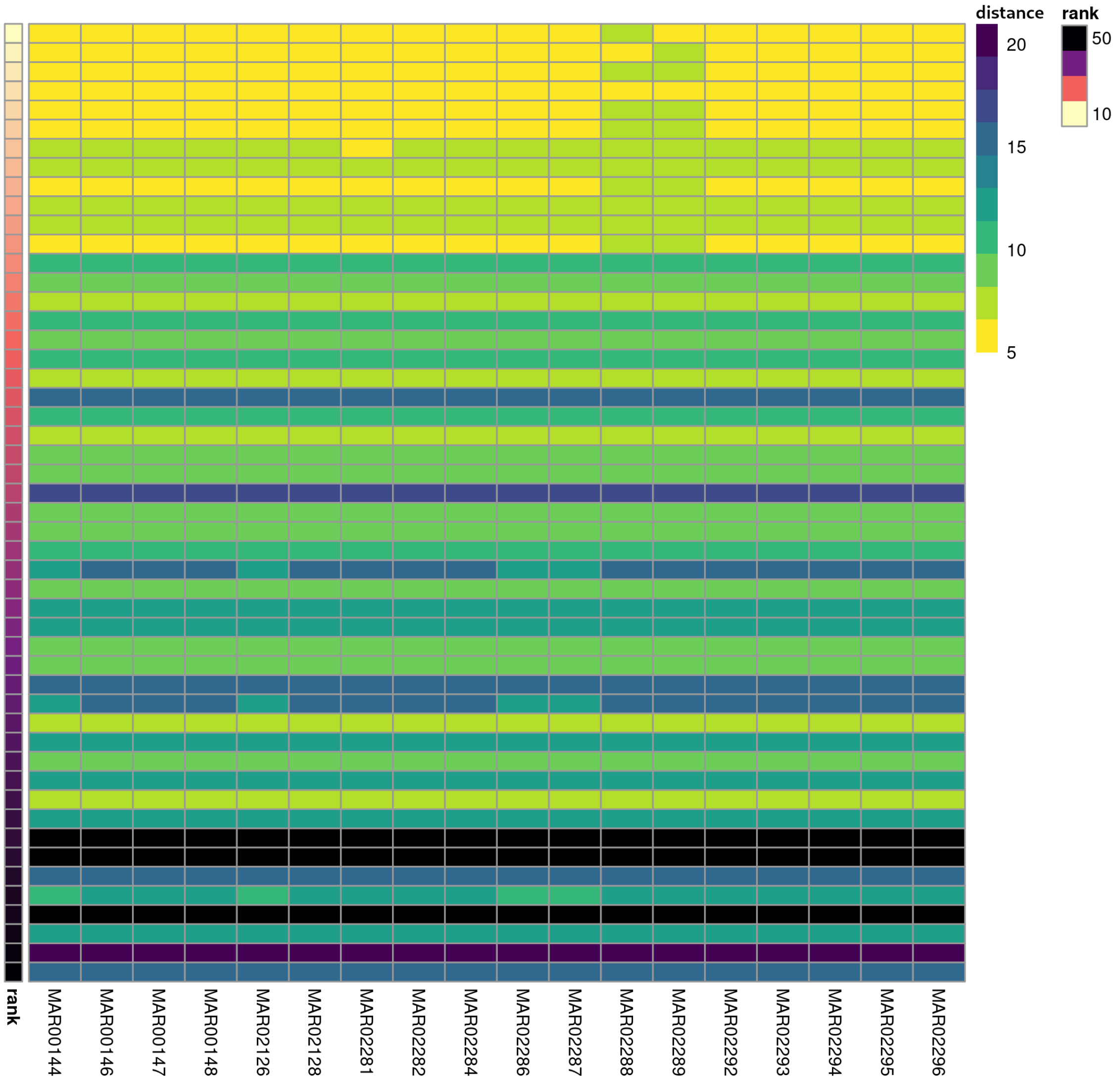

As expected, some top ranked metabolites are substrates or products of the altered reactions. We could also expect to find more distant repercussions across the network. We discovered through network analysis that more indirect relationships can be identified between altered reactions and top ranked metabolites. To do so, the metabolic network was first converted into a bipartite graph. A bipartite graph applied to metabolism consists of nodes as both metabolites and reactions, but the edges can only link different types: a reaction node can only be linked to metabolite nodes and vice versa.

The goal here is to calculate the distance between the reactions that were knocked-out in the SCD condition and the top 50 most changed metabolites according to SAMBA's predictions. The logical hypothesis is that the closer a metabolite is to the set of knocked-out reactions, the more

likely it is to be dysregulated by the disruption. In this section, calculating a distance between two entities using graph theory relied on the calculation of shortest paths. Fig I in S1 Text shows the distances from the 50 most changed metabolites (rows) as predicted by SAMBA for the SCD condition, in relation to the different reactions that were knocked-out (columns).

This distance heatmap shows that the highest ranked metabolites (top most rows, pale beige annotation on the left) are the closest (yellow) to the perturbed reactions, with distances of 5-6. As we move down the ranked list, the general trend is that the metabolites get further and further from the perturbed reactions, even reaching "infinite" distances, meaning that no path was found between the reactions (which is possible since the side compounds were removed, see Methods).

Fig J in S1 Text. Undirected subnetwork showing paths between the reactions affected by SCD (red squares) and the top 50 predicted most changed metabolites for this condition (coloured circles). Metabolites with a short path to the affected reactions are shown in yellow while darker colours correspond to a longer path.

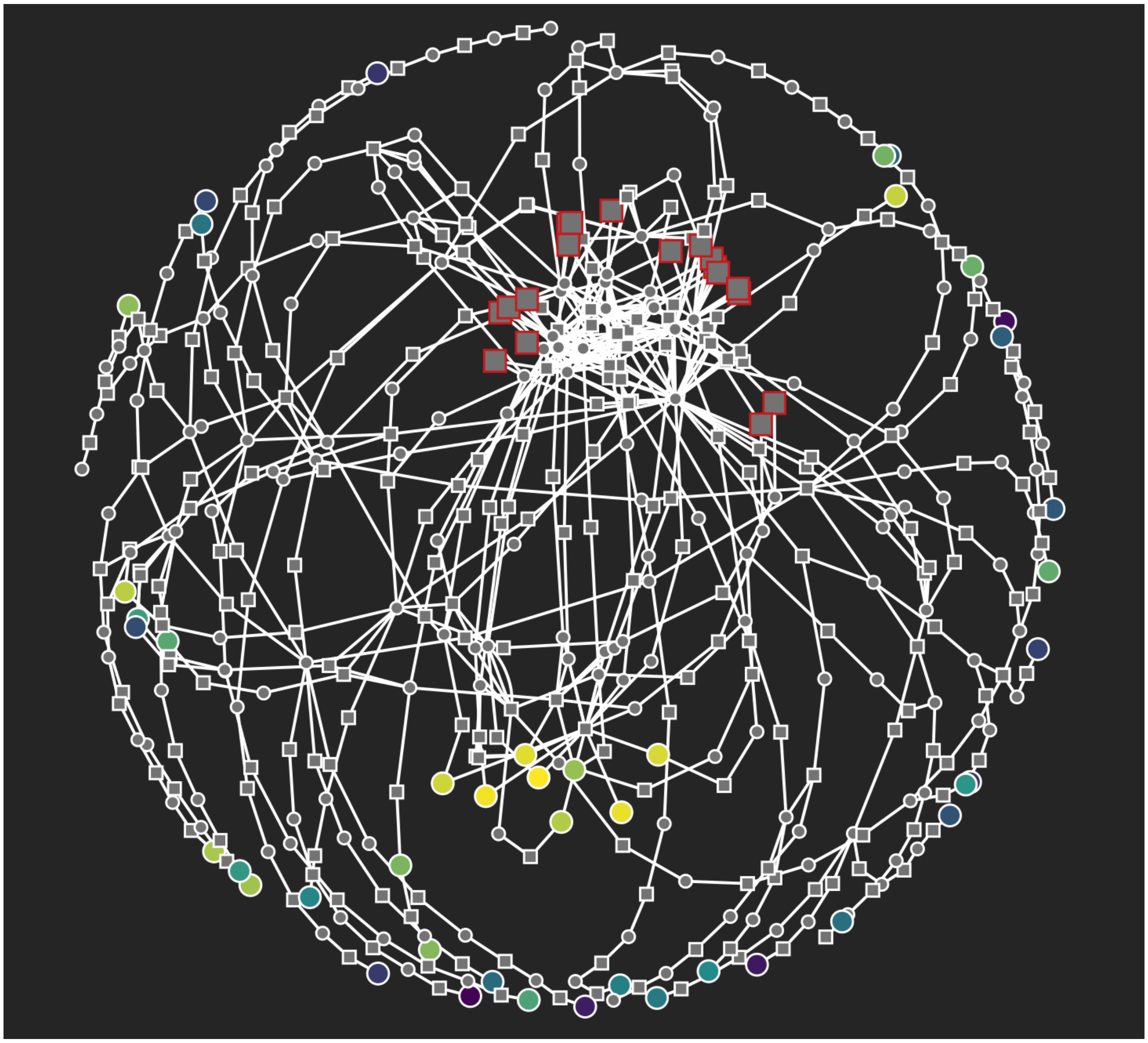

This data can also be represented on a metabolic network visualisation. Fig J in S1 Text displays the metabolic subnetwork extracted from Human1 using the perturbed reactions (red squares) and top 50 metabolites (coloured circles). Fig J in S1 Text shows that while the highly ranked metabolites are relatively close to the knocked-out reactions, they are not directly linked to them. Furthermore, many other affected metabolites can be found at a distance of 10 or more steps (shown as periphery metabolites in the circular layout of this figure). These are additional metabolites of interest that could be future paths for analysis which could not be directly inferred from the affected reactions and scenario. The far but highly ranked metabolites are metabolites we may not have thought of as potential metabolites of interest due to their distance from the disruption in the network, since they may seem unrelated at first glance.

For the distance calculation in this study, Human1 was converted to a bipartite graph using the Met4J library. Shortest paths were calculated after removing side compounds and on the undirected version of the graph (see following paragraphs).

In order to calculate distances correctly in a metabolic graph, side compounds must be removed. Side compounds create irrelevant links between reactions when the goal is to calculate how close a reaction is to another. A side compound is usually defined as a metabolite with a high degree of connectivity, i.e. involved in many reactions, and is not considered as the "main" substrate of the reaction. For example, ATP, ADP, and H_2O can be considered as side compounds. Unfortunately, defining a list of side compounds for a network is not an easy task as it can depend on the goal of the analysis as well as the organism or other parameters, and can even be a subjective choice. In this case, a manually curated list of side compounds was used with Human1 to remove edges that would shorten paths too much for the distance calculation to be of use.

The second parameter is choosing whether to use the directed or undirected version of the metabolic graph network. A directed network uses the directionality of each reaction in its catalysed state, whereas an undirected network removes this information when calculating a shortest path. Undirected paths represent the global distance of effect between two entities while directed paths are more indicative of upstream/downstream effects, which is why undirected paths were chosen for this study.

Fig K in S1 Text. ChemRich using top 54 metabolite sampling predictions for Fish-eye disease/ LCAT deficiency.

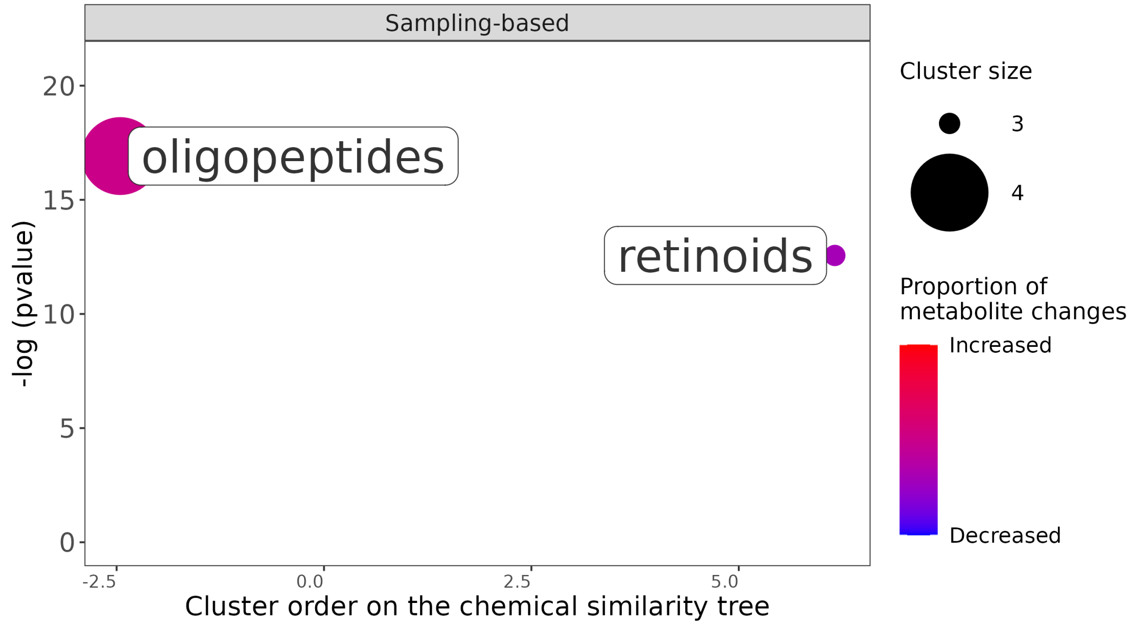

Table A in S1 Text. Table of the top 10 most differentially changed metabolites for the SCD gene KO using SAMBA. Human1 IDs correspond to the exchange metabolite IDs. Metabolites highlighted in blue were also significant in the -CoA 9-desaturase (SCD) metabolic Genome Wide Association Study (mGWAS) data.

Metabolite name (alternate name)	Human1 ID	CHEBI	SAMBA rank
9-Heptadecenoic acid	MAM01238e	80550	1
10-Heptadecenoate	MAM00003e	75094	2
gadoleic acid (eicosenoate)	MAM01235e	32419	3
heptadecanoic acid (margarate)	MAM02456e	32365	4
nonadecanoate	MAM02613e	39246	5
cis-tetradec-7-enoic acid	MAM00117e	53206	6
(5E)-tetradecenoyl-L-carnitine	MAM02974e	131957	7
prostaglandin A1	MAM02776e	15545	8
5-Tetradecenoic acid (physeteric acid)	MAM02745e	89393	9
Phytanic acid	MAM02746e	16285	10

Table B in S1 Text. ChemRich provides a metabolite-level table with each metabolite assigned to a cluster, the top 10 of which are shown in this table. These clusters are more specific than some of the annotations provided by ChEBI, namely those assigned to "long-chain fatty acid" by BiNChE are grouped into unsaturated and saturated fatty acids by ChemRich.

Compound Name	SMILES	Z-score	Cluster
9-heptadecylenic acid	CCCCCCC/C=C/CCCC CCCC(=O)O	−13.211	UnSaturated FA
(10Z)-heptadecenoic acid	CCCCCC\C=C/CCCCC CCCC(O)=O	−6.854	UnSaturated FA
9-eicosenoic acid	[H]\C(CCCCCCCCCC)= C(/[H])CCCCCCCC(O)=O	−4.342	UnSaturated FA
margaric acid	CCCCCCCCCCCCCCCC (=O)O	3.099	Saturated FA
nonadecylic acid	CCCCCCCCCCCCCCCCCC (=O)O	2.920	Saturated FA
(7Z)-tetradecenoic acid	CCCCCC/C=C\CCC CCC(=O)O	−2.387	UnSaturated FA
tetradecenoyl -carnitine(5)	CCCCCCCC/C=C/CCCC (=O)O[C@H](CC(=O)[O-]) C[N+](C)(C)C	−1.606	
prostaglandin A1	CCCCC[C@@H](/C=C/ [C@H]1C=CC(=O)[C@@H] 1CCCCCCC(=O)O)O	−1.400	prostaglandins a
physeteric acid	CCCCCCCC/C=C/CCCC (=O)O	−1.213	UnSaturated FA
Phytanate	CC(C)CCCC(C)CCCC(C) CCCC(C)CC(=O)O	−1.175	Saturated FA
